# Efficacy of Yukmijihwang-tang on symptoms of Alzheimer disease: A protocol for systematic review and meta-analysis

**DOI:** 10.1097/MD.0000000000026363

**Published:** 2021-06-25

**Authors:** Seunghee Lee, Do Hyung Kwon, Ju Yeon Kim, Yunna Kim, Seung-Hun Cho, In Chul Jung

**Affiliations:** a College of Korean Medicine, Daejeon University, Daejeon; b Department of Neuropsychiatry, College of Oriental Medicine, Kyunghee University, Seoul; c Research Group of Neuroscience, East-West Medical Research Institute, WHO Collaborating Center; d Department of Clinical Korean Medicine, Graduate School, Kyung Hee University, Seoul; e Department of Oriental Neuropsychiatry, College of Korean Medicine, Daejeon University, Daejeon, Republic of Korea.

**Keywords:** Alzheimer disease, dementia, protocol, systematic review, Yukmijihwang-tang

## Abstract

**Background:**

Alzheimer disease (AD) is the most common cause of dementia, which may lead to severe memory loss and other cognitive disorders. Yukmijihwang-tang (YMJ), a type of Korean traditional herbal medicine, has been shown to be effective against neurodegenerative diseases. Although a meta-analysis on the efficacy of YMJ on AD exists, the study had some limitations, and there have been several newly published studies assessing the effect of YMJ. Therefore, the purpose of this study is to evaluate the efficacy and safety of YMJ as a treatment for AD through a meta-analysis.

**Methods:**

A systematic search of the following electronic databases will be conducted to identify eligible studies: MEDLINE (PubMed), Elsevier (EMBASE), The Cochrane Central Register of Controlled Trials (CENTRAL), Cumulative Index to Nursing and Allied Health Literature (CINAHL), Korean Medical Database (KMBASE), Oriental Medicine Advanced Searching Integrated System (OASIS), Korean Traditional Knowledge Portal, Citation Information by NII (CiNii), China National Knowledge Infrastructure (CNKI). All randomized controlled trials assessing the efficacy and safety of YMJ on the symptoms of AD will be included. Two independent reviewers will perform article retrieval, deduplication, data screening, data extraction, quality evaluation, and data analyses using RevMan version 5.4. The Cochrane risk of bias tool will be used to assess the quality of the trials.

**Results:**

This study will provide synthesis of the cognitive function measured with neuropsychological tests, behavioral and psychological symptoms of dementia (BPSD), and activities of daily living (ADL) measured using validated scales. The clinical effective rate and adverse events will also be analyzed to assess the efficacy and safety of YMJ for treating AD.

**Conclusion:**

This systematic review will provide evidence for the efficacy and safety of YMJ in AD.

**Ethics and dissemination:**

Ethical approval is not required because individual patient data will not be included in this study. The study findings will be disseminated through conference presentations.

## 1 Introduction

Alzheimer disease (AD) is a type of brain disease that causes progressive neurodegenerative disorders.^[1]^ According to international classification of diseases, 11th edition, the onset is insidious with memory impairment typically reported as the initial presenting complaint. The level of cognitive functioning declines slowly and steadily from the previous level. Additional disabilities occur in cognitive domains (such as executive functions, attention, language, judgment and problem-solving abilities, psychomotor speed, and visuoperceptual or visuospatial functions) with disease progression.^[2]^ Also, AD is the leading risk factor for dementia.^[3]^ Dementia caused by AD often shows mental and behavioral symptoms such as depressed feelings and apathy in the disease's initial stages. At later stages, it may be accompanied by psychotic symptoms, confusion, irritability, agitation, changes in physical abilities (e.g., walking, sitting, and swallowing) and seizures.^[1]^

Currently, there is no cure to stop or slow down the destruction of neurons and treat AD completely. Therefore, drug and non-drug treatments are used to improve cognitive and behavioral symptoms of AD. Even though cholinesterase inhibitors (e.g., donepezil, rivastigmine, and galantamine) and N-methyl-D-aspartate receptor inhibitors (e.g., memantine) are currently the standard treatment for AD's cognitive symptoms,^[1,4]^ these drugs show limited efficacy and have been reported to have adverse events and drug tolerance development.^[1,4,5]^

In Korean medicine, the brain is thought to be generated by the kidney essence. Insufficiency of the kidney essence leads to a disorder of brain development and causes dementia.^[6]^ In the Dongeuibogam, Yukmijihwang-tang (YMJ) is stated to treat deficiency in the kidneys.^[7]^ YMJ is a Korean traditional herbal medicine composed of 6 herbal plants, including Rehmanniae radix preparata, Dioscoreae Rhizoma, Corni Fructus, Poria Sclerotium, Moutan Radicis Cortex, and Alismatis Rhizoma. Research on YMJ has been carried out for a long time and is still actively ongoing.^[8,9]^

Many studies have reported the pharmacological properties of YMJ on neurodegenerative diseases in both animal models and humans. In a recent experiment, YMJ normalized abnormal acetylcholine esterase (AChE) activity and ameliorated impairment of hippocampal memory ability due to chronic restraint stress in a mouse model.^[10]^ Also, YMJ had anti-amnestic effects and antioxidant defense system in scopolamine-induced memory impairment mouse model.^[11,12]^ A study that examined ordinary people, who did not have current or past neuronal or psychiatric disorders found the positive effect of YMJ on cognitive improvement and its potential in treating dementia patients with deficient cognitive ability.^[13]^ Additionally, a survey was conducted on general physicians and neuropsychiatrists of Korean medicine about herbal medicine treatment used on dementia. Based on the results, YMJ was rated as one of the most frequently used treatments in both groups.^[14,15]^ Therefore, through this study, we will investigate the safety and effect of YMJ on the symptoms of AD (such as cognitive function, behavioral and psychological symptoms of dementia [BPSD], and activities of daily living [ADL]).

## 2 Methods

### 2.1 Study registration

This protocol has been registered in the Open Science Framework (OSF), with the registration doi 10.17605/OSF.IO/BP578 (https://osf.io/bp578). The protocol will strictly follow the guidelines of Preferred Reporting Items for Systematic Reviews and Meta-Analyses protocols. The systematic review of this protocol will be developed by the Preferred Reporting Items for Systematic reviews and Meta-Analyses statement.

### 2.2 Inclusion and exclusion criteria for study selection

#### 2.2.1 Types of studies

We will include randomized controlled trials assessing the efficacy and safety of YMJ on the symptoms of AD regarding cognition, BPSD, and ADL.

#### 2.2.2 Types of participants

Participants diagnosed with AD will be included in the study. Studies involving vascular, frontotemporal, or other types of dementia will be excluded.

#### 2.2.3 Types of interventions and comparisons

Any formulation of YMJ will be included (such as granules, capsules, powder, and decoction). Its modified form will be included if the author has mentioned that it originated from YMJ. The studies will be included if YMJ was used with conventional treatment as an experimental intervention or compared with the same conventional treatment as a control group. Control interventions will include all types of control, such as placebo, conventional treatment, waiting-list control, or no treatment.

#### 2.2.4 Types of outcomes

The main outcomes will include cognitive function measured with neuropsychological tests (e.g., Mini-Mental State Examination, Hasegawa Dementia Scale, Montreal cognitive assessment , Alzheimer's Disease Assessment Scale-Cognitive Subscale), BPSD measured with validated scales (e.g., Neuropsychiatric Inventory, Behavioral Pathology in Alzheimer's Disease), and the activities of daily living measured with validated scales (e.g., ADL and Modified Barthel Index). Additional outcomes will be the clinical effective rate and adverse events.

### 2.3 Searching strategy

#### 2.3.1 Electronic searches

Two researchers will search the following electronic bibliographic databases to identify studies for the review: MEDLINE (PubMed), EMBASE, The Cochrane Central Register of Controlled Trials (CENTRAL), Cumulative Index to Nursing and Allied Health Literature (CINAHL), Korean Medical Database (KMBASE), Oriental Medicine Advanced Searching Integrated System (OASIS), Korean Traditional Knowledge Portal, CiNii, China National Knowledge Infrastructure (CNKI). The search will be restricted to studies written in English, Korean, Japanese, and Chinese. The search date for this review is from the inception to December 2020. The reference list of all identified relevant reviews and retrieved articles for additional studies will be reviewed. The exemplary search strategy for MEDLINE is shown in Table 1. According to the characteristics of each database, search terms may vary, and a comprehensive search will be conducted.

**Table 1:** PubMed search strategy.

#1	“Alzheimer Disease”[Mesh]
#2	alzheimer^∗^[Title/Abstract]
#3	AD[Title/Abstract]
#4	“Dementia, multi-Infarct”[Mesh]
#5	MCI[Title/Abstract]
#6	amnestic[Title/Abstract]
#7	(cognit^∗^ impair^∗^)[Title/Abstract]
#8	(cognit^∗^ declin^∗^)[Title/Abstract]
#9	(cognit^∗^ deficit^∗^)[Title/Abstract]
#10	(cognit^∗^ disturb^∗^)[Title/Abstract]
#11	“cognitive disorder^∗^”[Title/Abstract]
#12	(cognit^∗^ defect^∗^)[Title/Abstract]
#13	“Cognitive defect”[Title/Abstract]
#14	“Cognition Disorders”[Mesh]
#15	“Amnesia”[Mesh]
#16	“memory impairment”[Title/Abstract]
#17	CDR AND 0.5[Title/Abstract]
#18	“clinical dementia rating” AND 0.5[Title/Abstract]
#19	dement^∗^[Title/Abstract]
#20	“mild neurocognitive disorder”[Title/Abstract]
#21	“neurocognitive disorder”[Title/Abstract]
#22	“Subjective memory complaint^∗^”[Title/Abstract] OR SMC[Title/Abstract]
#23	#1 OR #2 OR #3 OR #4 OR #5 OR #6 OR #7 OR #8 OR #9 OR #10 OR #11 OR #12 OR #13 OR #14 OR #15 OR #16 OR #17 OR #18 OR #19 OR #20 OR #21 OR #22
#27	Yukmi^∗^[Title/Abstract] OR Yukmee^∗^[Title/Abstract] OR Yugmi^∗^[Title/Abstract] OR Yugmee^∗^[Title/Abstract] OR Yuk-mi^∗^[Title/Abstract] OR Yuk-mee^∗^[Title/Abstract] OR Yug-mi^∗^[Title/Abstract] OR Yug-mee^∗^[Title/Abstract] OR yukmijihwang^∗^[Title/Abstract] OR “yukmijihwang tang”[Title/Abstract]
#28	Liuwei^∗^[Title/Abstract] OR “Liu wei”[Title/Abstract] OR Liu-wei^∗^[Title/Abstract] OR “LWDHW herbal preparation”[Title/Abstract] OR “LD-Decoction”[Title/Abstract] OR “LW-AFC preparation”[Title/Abstract] OR “liuweidihuang tang”[Title/Abstract] OR liuweidihuang^∗^[Title/Abstract] OR “liuwei dihuang decoction”[Title/Abstract] OR (liuwei dihuang^∗^)[Title/Abstract]
#29	^∗^[Title/Abstract] OR ^∗^[Title/Abstract]
#30	Rokumi^∗^[Title/Abstract] OR TJ-87[Title/Abstract] OR rokumigan[Title/Abstract]
#31	“liu-wei-di-huang wan”[Mesh] OR “Liuwei Dihuang Decoction”[Mesh]
#32	yukmi[Mesh]
#33	Rokumigan[Mesh] OR TJ-87[Mesh]
#34	“LD-Decoction”[Mesh] OR “LW-AFC preparation”[Mesh]
#35	#27 OR #28 OR #29 OR #30 OR #31 OR #32 OR #33 OR #34
#36	#23 AND #35

### 2.4 Data collection and analysis

#### 2.4.1 Study selection

All retrieved articles according to the criteria described above will be imported to the reference management software Endnote 20.0.1 software (Clarivate Analytics, Boston, MA). After deduplication, 2 reviewers (SHL, DHK) will independently screen the titles and abstracts. The full text of the potentially eligible studies will be retrieved and independently assessed for eligibility by 2 reviewers (SHL, DHK). Any disagreement between the reviewers regarding the eligibility of the study will be resolved through discussion.

#### 2.4.2 Data extraction and management

The reviewers will extract the individual study's data using a prespecified data extraction form that includes study design, intervention, comparison, duration, follow-ups, outcome measures, results, and adverse events. We will enquire the original author for the missing data.

#### 2.4.3 Risk of bias assessment

Two independent reviewers will assess the quality of individual studies in accordance with the Cochrane risk of bias tool, whose items include the following:

- Random sequence generation
- Allocation concealment
- Blinding of participants
- Blinding of personnel
- Blinding of outcome assessment
- Incomplete outcome data
- Selective reporting
- Other bias.

Each risk of bias item will be rated as low, high, or unclear risk of bias.

#### 2.4.4 Data synthesis and analysis

The *I*^2^ test will be used to evaluate statistical heterogeneity. It will be classified as follows: low heterogeneity, for *I*^2^ under 25%; moderate heterogeneity, for *I*^2^ between 25% and 50%; and high heterogeneity, for *I*^2^ above 75%. Funnel plots will be used to assess potential reported bias when >10 trials are included in the study. For continuous data, mean differences with 95% confidence intervals will be used to pool the outcomes from the individual studies. When outcome measures do not correspond among studies, standardized mean difference will be used. Dichotomous data will be analyzed using relative risks (RRs) with 95% confidence intervals. Review Manager (RevMan, version 5.4, The Cochrane Collaboration, London, UK) will be used to conduct data synthesis.

## 3 Discussion

Dementia is one of the most common neurodegenerative diseases causing memory loss and cognitive impairment.^[16]^ Until 2018, it was estimated that 50 million people were living with dementia globally, and the number might triple by 2050.^[17]^ AD is estimated to cause 60% to 80% of dementia cases, but the cure does not exist in the present.^[1]^ The drugs currently used for treatment of symptoms are reported to have side effects and tolerance development. Therefore, it is crucial to identify an effective treatment for AD.

Herbal medicine has been widely used in many Asian countries for hundreds of years. YMJ is one of the general treatments used for various conditions, such as diabetes mellitus, neurosis, renal disorders, and osteoporosis, as well as to activate immune function and recover from weakness.^[13,18–21]^ Several studies have shown the efficacy of YMJ in improving the symptoms of AD. Although there is a previous meta-analysis about the effect of YMJ on dementia, the study included the literature on diverse kinds of dementia.^[22]^ In addition, some new clinical trials have been published thereafter. Therefore, it is necessary to conduct an updated systematic review and meta-analysis.

Following this protocol, we will strictly evaluate the quality of included studies on YMJ combined with or without conventional medicine in the treatment of AD. This systematic review will systematically and scientifically summarize the efficacy and safety of YMJ in AD.

## Acknowledgments

The authors would like to thank Editage (www.editage.co.kr) for English language editing.

## Author contributions

**Conceptualization:** In Chul Jung, Seung-Hun Cho.

**Data curation:** Seunghee Lee, Do Hyung Kwon.

**Formal analysis:** Seunghee Lee, Do Hyung Kwon.

**Funding acquisition:** In Chul Jung, Seung-Hun Cho.

**Writing – original draft:** Seunghee Lee, Do Hyung Kwon, Ju Yeon Kim.

**Writing – review & editing:** Seunghee Lee, Do Hyung Kwon, Yunna Kim, In Chul Jung, Seung-Hun Cho.
